# Personal protective equipment (PPE) related adverse skin reactions among healthcare workers at the main COVID-19 isolation center in Barbados

**DOI:** 10.3389/fpubh.2022.978590

**Published:** 2022-10-11

**Authors:** Ambadasu Bharatha, Kandamaran Krishnamurthy, Damian Cohall, Sayeeda Rahman, Corey A. Forde, Rhea Corbin-Harte, Nkemcho Ojeh, Russell Kabir, Ali Davod Parsa, Ahbab Mohammad Fazle Rabbi, Md Anwarul Azim Majumder

**Affiliations:** ^1^Faculty of Medical Sciences, The University of the West Indies, Cave Hill Campus, Bridgetown, Barbados; ^2^PICU Consultant, Queen Elizabeth Hospital, Bridgetown, Barbados; ^3^American University of Integrative Sciences, School of Medicine, Bridgetown, Barbados; ^4^Infection Prevention and Control/Infectious Diseases Programs, The Queen Elizabeth Hospital, Bridgetown, Barbados; ^5^Isolation Facilities Client Relations and Activities, Bridgetown, Barbados; ^6^Faculty of Health Education, Medicine and Social Care, Anglia Ruskin University, Chelmsford, United Kingdom; ^7^Faculty of Health, Education, Medicine and Social Care, Anglia Ruskin University, Cambridge, United Kingdom; ^8^Department of Population Sciences, University of Dhaka, Dhaka, Bangladesh

**Keywords:** COVID-19, PPE, adverse skin reactions, healthcare workers, Barbados

## Abstract

**Background:**

The use of personal protective equipment (PPE) reduces the risk of transmission of infectious agents significantly among healthcare workers (HCWs). The study aimed to investigate the prevalence and characteristics of PPE-related adverse skin reactions among HCWs working at the main COVID-19 isolation center in Barbados.

**Methods:**

A cross-sectional web-based online survey was conducted during April to June 2021 which recorded demographic information, details of PPE use and adverse skin reactions including severity and duration of onset of symptoms.

**Results:**

Most of the respondents used PPE for consecutive days (77.9%), 1–6 h/day (59.2%), and more than a year (62.5%). Fewer than half of the participants (45.6%) experienced adverse skin reactions from the use of PPE. The reactions were mostly observed in the cheeks (40.4%) and nose bridges (35.6%). Females had more reactions than their male counterparts (*p* = 0.003). The use of N95 masks and a combination of surgical and N95 masks produced adverse effects predominantly in the ears (60%) and cheeks (56.4%). Binary logistic regression showed that female HCWs (OR = 5.720 95% CI: 1.631, 20.063), doctors (OR = 5.215 95% CI: 0.877, 31.002), and longer duration of PPE use (>1 year) (OR = 2.902 95% CI: 0.958, 8.787) caused a significantly higher prevalence of adverse skin reactions.

**Conclusion:**

The PPE-related skin reactions were common among HCWs which mainly occurred due to prolonged use. Preventive measures inclusive of appropriate training of HCWs on the use of PPE are recommended to minimize these adverse events.

## Introduction

Frontline healthcare workers (HCWs) have faced numerous challenges while treating and managing COVID-19 patients during the pandemic (1–5). These frontline HCWs are susceptible to infection, and they account for 10% of the COVID-19 cases (1). During the Severe Acute Respiratory Syndrome (SARS) and Middle East Respiratory Syndrome (MERS) outbreaks, a quarter of infected cases were among HCWs (6, 7). Studies showed that the use of personal protective equipment (PPE) could reduce the risk of infection significantly among HCWs (8, 9). Standard measures such as the use of gloves, gowns, and eye protection are recommended by the Centers for Disease Control and Protection (CDC) (10) and the World Health Organization (WHO) (11). Furthermore, the CDC recommends the use of N95 filtering facepiece respirators by all COVID-19 patients (both suspected and confirmed). Also, the WHO recommends the use of surgical masks by HCWs providing care to COVID-19 patients along with the exclusive use of respirators by HCWs for aerosol-generating procedures (12).

The mode of transmission of COVID-19 has been shown to be respiratory droplets and contact with fomites. The use of PPEs is critical to reducing cross-transmission of the infection (6–8). The PPE provides a layer of safety for HCWs by limiting contact between clinical staff and patients (13). HCWs require appropriate PPE training and consistent guidance to protect their health and well-being (14). Several studies have already highlighted the high frequency of physical health issues, including skin abnormalities due to PPE use among HCWs (15–17). These are mainly due to the long-term wearing, inappropriate re-use, ill-fitting PPE and PPE shortages during the COVID-19 pandemic (16–19). Moreover, PPE may generate a series of skin lesions due to (i) long-term sealing caused by poor air permeability, (ii) friction-induced skin conditions such as erythema, blisters or ulcers, associated with pain and even secondary infection, and (iii) pressure on the skin (20, 21). There is an increasing incidence of occupational dermatoses due to facial PPE, including adverse cutaneous reactions, irritant contact dermatitis, allergic contact dermatitis, acneiform eruptions, and contact urticaria (22). Appropriate strategies need to be taken to prevent PPE-related adverse events by supplying an adequate number of PPEs and organizing training on the proper use of PPE by HCWs (16, 19).

The first COVID-19 case was identified in Barbados on 17 March 2020 (2). As of Jan 09, 2022, 11% of the Barbadian population tested positive for COVID-19, and the death rate was reported at 0.93 per 1,000 people (23). The aim of this study is to investigate the prevalence and characteristics of adverse facial skin reactions due to the use of PPE among HCWs working in a COVID-19 isolation center in Barbados and suggest potential risk factors and management strategies for these reactions.

## Methods

### Setting

Barbados is the most southeastern island in the Caribbean and spans an area of 432 km^2^ (166 sq mi) with a population of 287,000. The Queen Elizabeth Hospital (QEH) is the only tertiary care hospital on the island with a bed capacity of 519. Four isolation centers were established to combat the COVID-19 pandemic as the caseload on the island increased: Harrison Point, Enmore Center, Psychiatric Hospital and the Sunbay Hotel. The largest isolation facility is the Harrison Point, which was established in a refurbished military base with a capacity of 200 beds. The facility has been staffed with 30 nurses, 18 physicians, 45 housekeeping and 18 orderlies and subdivided into primary, secondary and tertiary care units. The tertiary care unit accommodates asymptomatic but confirmed COVID positive cases, the secondary unit admits mild cases where patients require oxygen for management, and moderate to severe cases are managed in the primary care unit. The Enmore Center is a 3-bedded intensive care unit located just opposite the QEH. Patients at this site are transferred to the Harrison Point isolation center once they are stabilized. The Enmore Center is staffed with 10 nurses, 2 physicians and 4 housekeeping staff to cover the 24 h schedule at the facility. The other two isolation centers are the Psychiatric Hospital and the Sunbay Hotel where mild cases are managed. Each center is staffed with ~15 nurses, 4 physicians, 4 orderlies, and 8 housekeeping staff.

### Study design, sampling and data collection

The study used purposive sampling of all HCWs working at the COVID-19 main isolation center in Barbados. HCWs were invited to complete a cross-sectional online survey assessing adverse skin reactions using PPE. Inclusion criteria for the study are staff working in areas deemed necessary for PPE usage, namely staff caring for suspected and infected COVID-19 patients or staff at the frontline hospitality services, receptionists and ushers. Email invitations were sent with a Google Forms survey link from 1 April 2021 to 21 June 2021.

The study used a validated questionnaire developed by Abiakam et al. (17). The modified questionnaire was pretested, and the final version was approved for use to conduct the survey. Items were grouped in the following sections: (i) demographic information, (ii) occupational related information, and (iii) adverse skin reactions (Supplementary material). To assess the effect of PPE on the skin, two different measures were recorded for each participant. The respondents' perception of their skin health was assessed prior to and after the use of PPE by self-reports. The measurement of pain due to the use of PPE was recorded on a scale of 0 (no pain) to 10 (highest pain). The perceived skin health before and after PPE use was assessed by a Likert scale of 1 (The worst skin health you can imagine) to 10 (The best skin health you can imagine).

Participation was purely voluntary and informed consent was implied by completing the questionnaire.

### Ethical approval

The study was approved by the University of the West Indies, Cave Hill Campus/Barbados Ministry of Health and Wellness Research Ethics Committee/Institutional Review Board (IRB No. 210322-B).

### Statistical analysis

Demographic data and some data on adverse effects due to the use of PPE were analyzed using descriptive statistics mainly. Frequency distributions were obtained to analyze the incidence and prevalence of skin reactions that occurred due to PPE use during the period of observation. Bivariate correlations were examined between the average duration of PPE use (also with the history of using PPE), demographic characteristics, and different skin effects of PPE. To assess the significant association between two variables, chi-square statistics with *p*-values were calculated. All statistical analysis was performed using IBM SPSS 20.0.

## Results

### Demographic characteristics

Out of 215 HCWs, 104 completed the survey and the response rate was 48.4%. The majority of the respondents were females (71.2%) and the nurses represented the largest group (45.2%) of HCWs. The majority of the study respondents were aged between 25 and 34 years (41.3%) (Table 1). In addition, more than half of the respondents (52.4%) were employed in public /government institutes. Eighty percent of the respondents reported that they had no chronic diseases. Only 40.4% of respondents attended a PPE fit testing appointment. Most of the respondents felt comfortable (86.5%), safe (99%), and able to breathe easily (82.5%) while using PPE (Table 2). Gender specific influence on the use of protective equipments is shown in Table 3.

**Table 1:** Sociodemographic variables and prevalence of adverse effect in skin/face due to PPE use.

**Variable**	**Total** **respondents**	**Adverse skin reactions**		***p*** **-value**
		**Yes (47)**	**No (56)**	
**Age**				
18–24 years	6 (5.8%)	3 (6.4%)	3 (5.4%)	
25–34 years	43 (41.3%)	21 (44.7%)	22 (39.3%)	
35–44 years	35 (33.7%)	16 (34%)	18 (32.1%)	0.883
45–54 years	17 (16.3%)	6 (12.8%)	11 (19.6%)	
55+ years	3 (2.9%)	1 (2.1%)	2 (3.6%)	
**Gender**				
Male	30 (28.8%)	7 (14.9%)	23 (41.1%)	0.003
Female	74 (71.2%)	40 (85.1%)	33 (58.9%)	
**Profession**				
Nurse	47 (45.2%)	23 (48.9%)	24 (42.9%)	
Doctor	16 (15.4%)	10 (21.3%)	6 (10.7%)	
Orderly	13 (12.5%)	2 (4.3%)	11 (19.6%)	
Housekeeping	19 (18.3%)	8 (17%)	10 (17.9%)	0.225
Medical staff	5 (4.8%)	2 (4.3%)	3 (5.4%)	
Administrative staff	4 (3.8%)	2 (4.3%)	2 (3.6%)	
**Time since started using PPE**				
0–3 months	10 (9.6%)	2 (4.3%)	8 (14.3%)	
3–6 months	14 (13.5%)	4 (8.5%)	10 (17.9%)	
6–9 months	9 (8.7%)	6 (12.8%)	3 (5.4%)	0.052
9–12 months	6 (5.8%)	1 (2.1%)	5 (8.9%)	
12+ months	65 (62.5%)	34 (72.3%)	30 (53.6%)	
**Daily average Duration of using PPE**				
1–6 h	61 (59.2%)	24 (51.1%)	37 (66.1%)	
6–8 h	26 (25.2%)	14 (29.8%)	11 (19.6%)	
8–10 h	6 (5.8%)	4 (8.5%)	2 (3.6%)	0.453
10–12 h	7 (6.8%)	3 (6.4%)	4 (7.1%)	
12–13 h	3 (2.9%)	2 (4.3%)	1 (1.8%)	

**Table 2:** Use of protective equipment by the respondents (*n* = 104).

**Variable**	**Number of respondents (%)**
**Average number of working days/week**	
3 days	2 (1.9%)
4 days	68 (66.0%)
5 days	23 (22.3%)
6 days	5 (4.8%)
7 days	5 (4.8%)
**Eye protection instrument**	
General safety glasses	1 (1.0%)
Chemical splashing goggles	1 (1.0%)
Face shield	102 (98.0%)
**Type of mask used by the respondent**	
Surgical mask	43 (41.3%)
N95	39 (37.5%)
KN95	2 (1.9%)
Both	20 (19.2%)
**Attended PPE fit testing appointment**	
Yes	42 (40.4%)
No	62 (59.6%)
**Comfort of using PPE**	
***Feeling comfortable***	
Yes	90 (86.5%)
No	14 (13.5%)
***Breathing easily***	
Yes	86 (82.7%)
No	18 (17.3%)
***Feeling safety on PPE***	
Yes	103 (99%)
No	1 (1%)

**Table 3:** Gender specific influence on the use of protective equipments by (*n* = 103).

**Sex**	**Profession**	**Adverse skin reactions**		**Chi-square** **(χ2)**
		**Yes (47)**	**No (56)**	
Male	Nurse	0 (0%)	2 (6.7%)	8.571
	Doctor	4 (13.3%)	2 (6.7%)	
	Orderly	2 (6.7%)	10 (33.3%)	
	Housekeeping	1 (3.3%)	5 (16.7%)	
	Medical staff	0 (0%)	3 (10%)	
	Administrative staff	0 (0%)	1 (3.3%)	
Female	Nurse	23 (31.5%)	22 (30.1%)	3.449
	Doctor	6 (8.2%)	4 (5.5%)	
	Orderly	0 (0%)	1 (1.4%)	
	Housekeeping	7 (9.6%)	5 (6.8%)	
	Medical staff	2 (2.7%)	0 (0%)	
	Administrative staff	2 (2.7%)	1 (1.4%)	

### PPE usage

More than three-quarters of the respondents (77.9%) used PPE for consecutive days, more than half (59.2%) used it for 1–6 h/day and approximately two-thirds used it for more than a year (62.5%) (Table 1). The average number of working days per week reported by the respondents was 4. In relation to protective equipment, 98% used a face shield as an eye protection instrument. Majority of the respondents (41.3%) reported that they used the surgical mask, followed by HCWs using N95 masks (37.5%) and those using both surgical and N95 masks (19.2%) (Table 2).

### Face skin health

The use of PPE caused red lesions (35.9%), indentation lesions (36.5%), and broken skin (2.9%) in facial areas. More than 35% of respondents stated that they had the best skin health prior to using PPE, while 28.1% had the same skin health after PPE use. On a scale of 1 (worst) to 10 (best), ~24 and 32% scored between 1 and 5 to indicate their skin health before and after using the PPE, respectively.

### Skin reactions at the various facial locations

More than 45% (*n* = 47) of participants experienced adverse skin reactions from the use of PPE. Redness, itchiness, rash, pressure damage and dry skin were the different reactions reported by the respondents. These occurred specifically at five face locations, namely the forehead, the nose, cheeks, ears and under lips as summarized in Table 4. The use of face masks caused skin reactions mainly at the cheeks (40.4%), nose (35.6%), ears (34.6%), and under lips (19.2%). The respondents who were using a face shield as eye protection suffered skin reactions on the forehead (34.6%).

**Table 4:** Different levels of skin problems in different parts of the face due to using PPE.

**Problems**	**Forehead**	**Nose**	**Cheeks**	**Under lips**	**Ears**
**Skin reactions**	***n*** **−36**	***n*** **−37**	***n*** **−42**	***n*** **−20**	***n*** **−36**
Redness	15 (41.7%)	11 (29.7%)	14 (33.3%)	7 (35%)	12 (33.3%)
Itchiness	3 (8.3%)	3 (8.1%)	2 (4.8%)	1 (5%)	6 (16.7%)
Rash	2 (5.6%)	4 (10.8%)	8 (19%)	4 (20%)	0%
Pressure damage	6 (16.7%)	9 (24.3%)	6 (14.3%)	3 (15%)	14 (38.9%)
Dry skin	0%	1 (2.7%)	1 (2.4%)	1 (5%)	0%
Both redness and pressure damage	7 (19.4%)	6 (16.2%)	5 (11.9%)	1 (5%)	0%
Itchiness and rash	2 (5.6%)	0%	1 (2.4%)	1 (5%)	0%
Rash and dry skin	0%	0%	1 (2.4%)	0%	2 (5.6%)
All of the mentioned effects together	1 (2.8%)	3 (8.1%)	4 (9.5%)	2 (10%)	2 (5.6%)
**No skin reactions**	***n*** **−104**				
	68 (65.4%)	67 (64.4%)	62 (59.6%)	84 (80.8%)	68 (65.4%)

**Figure 1:**
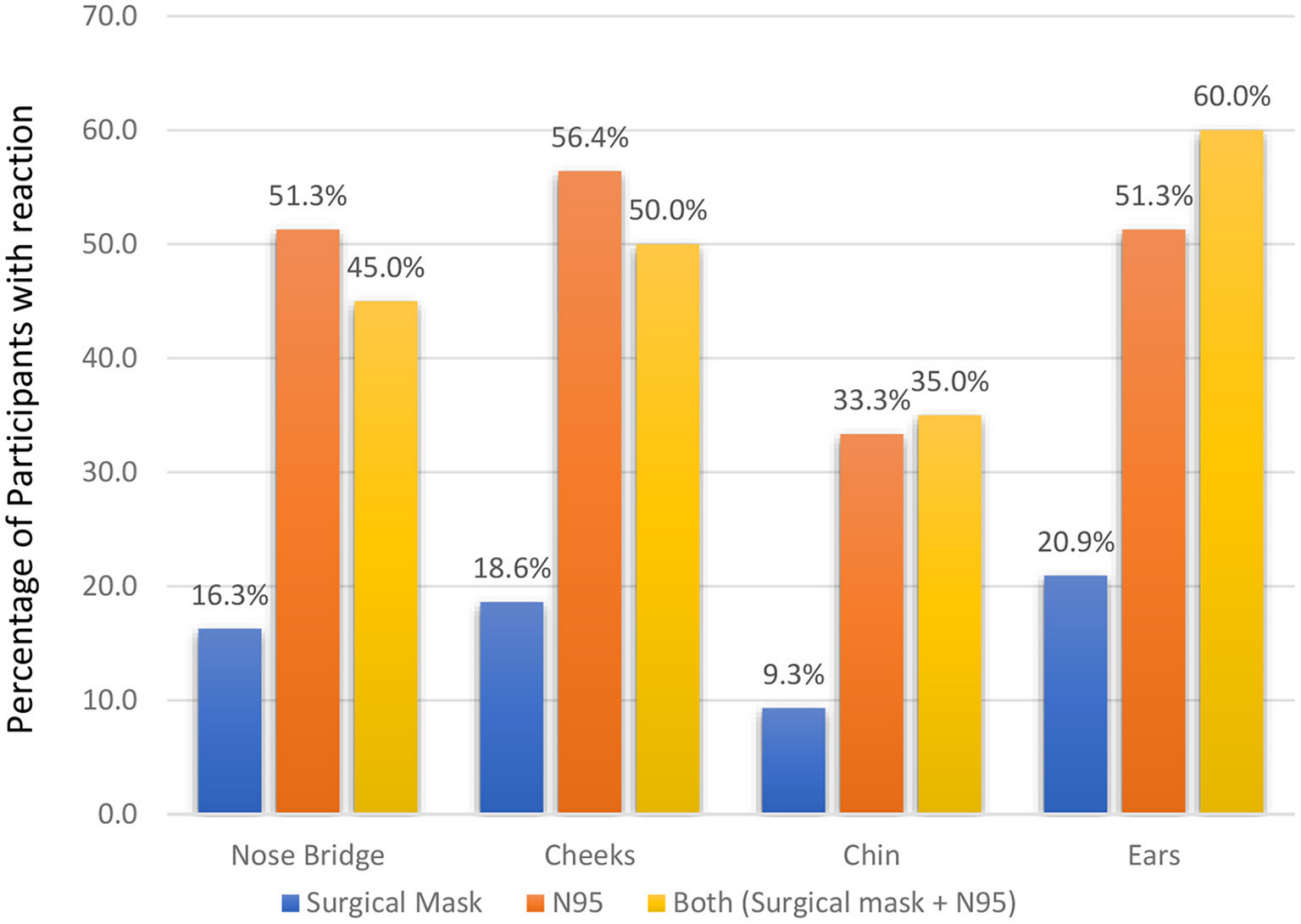
Type of mask used and incidence (%) of adverse skin reactions.

**Figure 2:**
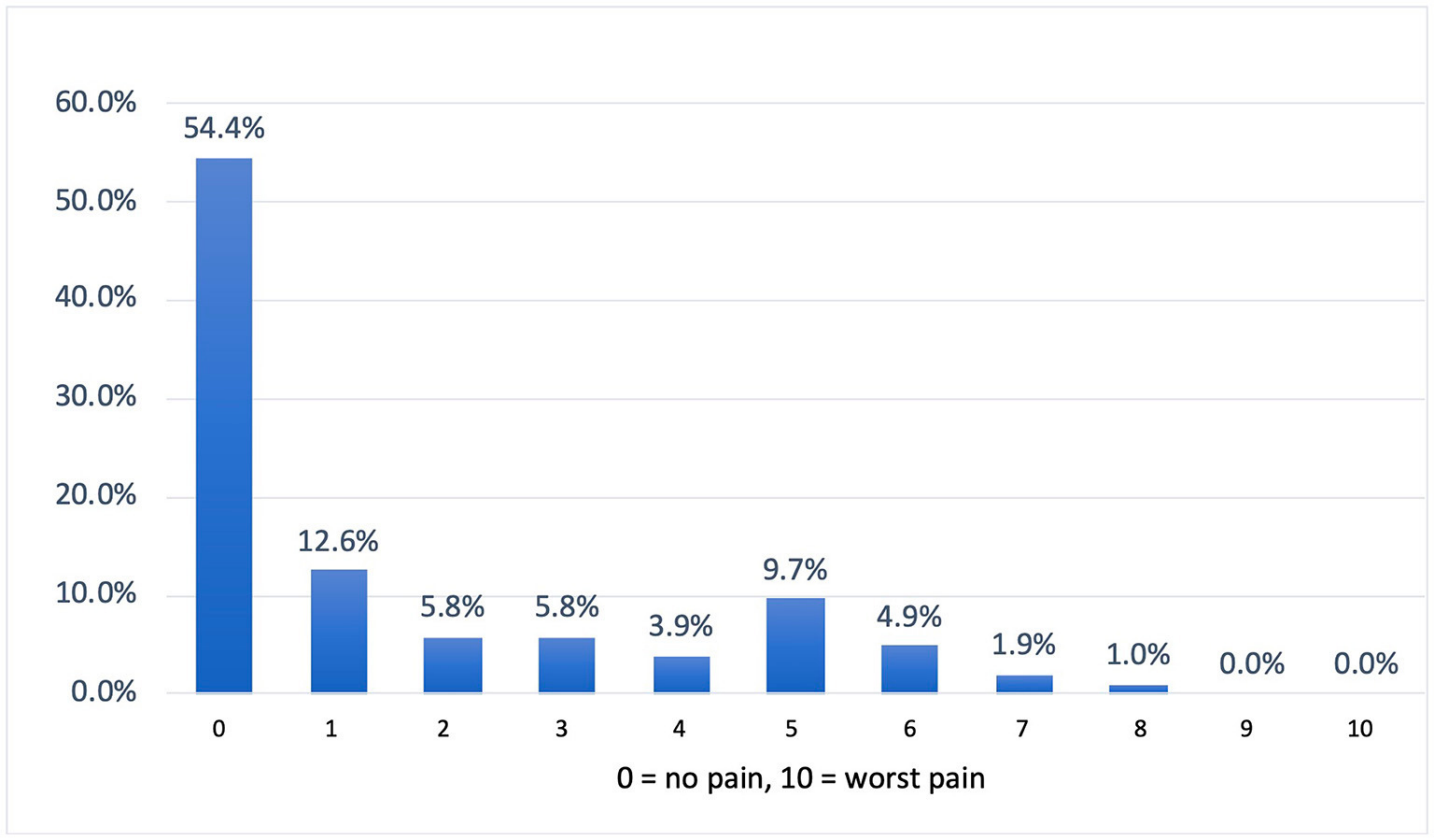
Distribution of pain measures.

Female professionals had more adverse outcomes for using PPE than their male counterparts with a significant statistical association (*p* = 0.003). More than half of the respondents reported that their cheeks (56.4%), nose bridge (51.3%) and ear (51.3%) were affected by using N95 masks (Figure 1). The use of both surgical masks and N95 masks produced adverse effects predominantly at the ears (60%) and cheeks (50%). When asked about the overall pain due to the use of PPE in a scale of 0 (no pain) to 10 (highest pain), more than half of respondents (54.4%) reported no pain (Figure 2).

### Determinants of adverse skin reactions due to use of PPE

To check whether various demographic variables and use pattern of PPE have any effect on the skin, we used binary logistic regression. The findings are summarized in Table 5. Here the dependent variable is whether the HCWs have any adverse effect on the skin due to using PPE. Gender, profession, and duration of PPE use were found to have a significant adverse skin reaction. Female HCWs were 5.7 times more likely to have adverse skin reactions than their male counterpart. Doctors are 5.2 times more risk than the nurses to develop adverse reactions. The use of PPE for longer was also found to be significant in this context, those who are using PPE for more than 1 year were 2.9 times more likely to have adverse skin reactions than those who were using less than a year.

**Table 5:** Logistic regression coefficients and odds ratios (95% CI) for determinants adverse reactions.

**Variable**	**OR (95% CI)**
**Age**	
<35 years (ref)	
≥35 years	0.613 (0.197, 1.902)
**Gender**	
Male (ref)	
Female	5.720^***^ (1.631, 20.063)
**Profession**	
Nurse (ref)	
Doctor	5.215^*^ (0.877, 31.002)
Others	1.569 (0.399, 6.163)
**Average number of working days/week**	
<5 days (ref)	
≥5 days	1.965 (0.726, 5.317)
**Type of mask used by the respondent**	
Surgical mask (ref)	
N95	1.358 (0.448, 4.4117)
Both	0.599 (0.162, 2.209)
**Time since started using PPE**	
<12 months (ref)	
≥12 months	2.902^*^ (0.958, 8.787)
**Daily average Duration of using PPE**	
<6 h (ref)	
≥6 h	1.141 (0.442, 2.947)

Reference category is denoted by (ref). Significance:

*** p < 0.01,

* p < 0.1.

## Discussion

This study was conducted in Harrison's Point, the main COVID-19 isolation center of Barbados, to assess facial skin reactions due to the use of PPE by HCWs. Less than half of the participants (45.6%) reported changes in skin health as a direct result of PPE usage. A systematic review analyzed 14 studies (*n* = 11,746 HCWs) from 16 countries and reported that the prevalence of skin changes due to the use of PPE among HCWs was 78% with a range from 42.8 to 95.1% reported among the studies (15). The causes of a wide range of prevalence include the use of PPE for varied periods due to the PPE demand and supply challenges, the increased workload of HCWs, lack of training and awareness regarding PPE usage, and not wearing (donning/doffing) PPE in the appropriate way (24–26). Our study found that doctors had a higher risk of developing adverse skin reactions compared to nurses. This may be attributable to prolonged wear of PPE by doctors due to longer clinical shift work and on-call hours. We also found that female professionals suffered more skin reactions when using PPE than their male counterparts (*p* = 0.003) and they were 5.7 times more likely to have adverse skin reactions than their male counterparts (OR = 5.720 95% CI: 1.631, 20.063). Our finding is consistent with the findings of some recent studies conducted during the COVID-19 pandemic (21, 27–29). More skin reactions among females may be explained by genetic factors, hormonal differences, physical activity, hygiene practices and the use of skin care products (15). Moreover, PPE is typically designed for white male size and shape and incorrect fitting for the anthropometric facial features of Black, Asian, and Minority (BAME) ethnicities has been reported elsewhere (17, 30). Hence, in this study, ill-fitted PPE, due to gender differences in facial features, may account for the increased skin reactions seen among females when worn for extended periods.

The current study found compelling evidence that the use of PPE for prolonged periods without skin relief is linked to the occurrence of adverse skin reactions. Our findings also demonstrated that those HCWs using PPE for a longer duration (>1 year) may be more likely to suffer adverse skin reactions, than those who were using less than a year. The majority of the respondents in our study who used PPE for more than a year, consecutive days or an average of 4 days per week and 1–6 h per day suffered skin reactions. The most commonly affected areas were the cheeks and the nose (Table 3), which is consistent with previous findings (29, 31–33). The use of PPE for longer periods causes compression injuries and excessive sweating; both stimulating the skin reactions in the above-mentioned body areas (31, 34–36). The use of PPE also causes skin barrier dysfunction due to prolonged contact time or increased frequency of use (37, 38). Specifically, prolonged exposure of PPE to the skin surface can cause pressure, friction and shear forces which can inflict skin and underlying tissue damage. Moreover, the combination of excessive sweating and moisture compromises the epidermal stratum corneum and reduces the mechanical load tolerance of the skin thus compromising the barrier function of the skin and providing grounds for injury (17) and secondary infection (39). Profuse sweating can also induce skin itching, pain and redness (15). Pressure damage to the skin behind the ears can be the result of repetitive friction attributable to the face masks string around the ears (16). The itching experienced may be due to the humid microenvironment that is generated under the PPE along with discomfort after extended PPE usage. The mask material may also trigger an allergic reaction which can give rise to contact dermatitis (21). A systematic review conducted by Keng et al. (39) using sixteen studies (*n* = 3,958) found that the most affected sites were nose or nose bridge (24.7%) and cheeks (21.3%). Another systematic review (16 studies) identified irritant contact dermatitis due to pressure and friction which were common on the cheeks and nasal bridge (40). Several studies showed that HCWs who wore PPE more than 6 h daily were at increased risk of adverse skin reactions (31, 37, 41).

It was found that wearing an N95 mask and a combination of both surgical and N95 masks were associated with a higher incidence of adverse skin reactions than wearing a surgical mask (Figure 1). Battista et al. noted that the incidence of adverse skin reactions was higher in the healthcare staff wearing N95 masks (41). Another study by Hu et al. (21) also reported a higher incidence (95.1%) of the skin reactions provoked by using an N95 mask. An Indian study recorded adverse skin reactions in the nasal bridge (76.64%) and ears (66.42%) after wearing N95 masks (16). Similar findings were echoed in China (21) and Singapore (42) among nurses and other HCWs, respectively. The skin reactions of N95 masks occur as HCWs have to tie the mask tightly using the metal clip to protect themselves from the COVID-19 infection (16). This may cause physical problems (e.g., headache, nausea, vomiting, etc.) due to hypoxemia and hypercapnia (43) especially in the warm and humid climate prevailing in the Caribbean countries (44). In addition, the combination of heat, humidity and moisture generated under N95 masks along with prolonged pressure and friction on the skin surface creates a microenvironment that raises the risks of skin reactions (45).

Only 40.4% of study respondents reported that they attended the PPE fit testing. Although most respondents reported being comfortable, safe and able to breathe easily while wearing PPE, a narrative review (46) states that fit testing is recommended by various international and national bodies to ensure respirators fit appropriately for the individual HCW and also to avoid transmission rates. Fit testing is also important as a training measure and can improve the mental wellness of HCWs. One study demonstrated a correlation between mental health and the number of skin reactions caused by the use of PPE (47). Furthermore, training also reduces the occurrence of adverse skin reactions and improves mental/physical health and boosts the morale and quality of life of HCWs (48, 49). Long-term studies should be conducted to evaluate the burnout, anxiety, depression, and emotional effects due to the use of the PPE and problems experienced by the HCWs providing patient care. We also found that there was a 20% reduction in the HCWs' perception of their facial skin health after their use of PPE. Adequate skin care before and after using PPE and applying barrier protectors and moisturizers on a regular basis is recommended (50).

Various studies suggest that HCWs should perform regular skin checks during and between periods of wearing PPE (17). HCWs wearing PPE should maintain hygiene and apply skin protectors or moisturizers to the contact areas with the PPE (50). After applying PPE, check for a “good” fit and ensure there are no areas of extra pressure. To relieve pressure, persons should provide frequent relief from PPE when safe, especially during long clinical shifts (51). As soon as adverse reactions are observed, switching the PPE device is recommended to avoid exposing vulnerable skin sites (17). It is reassuring that approximately three-quarters of the respondents in our study had consented to monitor their skin health on a weekly basis.

### Limitations

One of the important limitations of the present study was the small sample size and the use of HCWs in a single isolation center which may impact generalizability, as the study did not include other isolation or healthcare centers (selection bias). In addition, “recall” and “answer” biases cannot be ruled out as HCWs responded to a self-administered questionnaire and provided self-reported assessments of adverse reactions. Previous skin problems were not explored prior to the study nor were formal verification or diagnoses of the skin reactions ascertained in this study. Therefore, the factors associated with the causes and severity of these adverse effects were not assessed independently. Additionally, the present study failed to appraise the HCWs' psychological effects, quality of life and morale due to PPE induced skin problems. As this is the first study of its kind in the Caribbean region, it sets the scene to investigate the incidence of adverse skin reactions which can be caused by the long-term and inappropriate use of PPE by the HCWs.

## Conclusion

The study identified several PPE-related skin reactions which were common among HCWs working in a COVID isolation center in Barbados. These were induced due to prolonged and inappropriate use of PPE. The cheeks and nasal bridge were the most affected areas and female HCWs were more susceptible to adverse effects than males. HCWs who wore an N95 mask or a combination of surgical and N95 masks had a higher rate of adverse skin reactions than those who wore a surgical mask alone. To reduce the risk of skin reactions of frontline HCWs, there is an urgent need to improve PPE guidelines and the design/materials used to manufacture protective equipment. Moreover, preventive measures and appropriate training to counter these adverse events are recommended.

## Data availability statement

The raw data supporting the conclusions of this article will be made available by the authors, without undue reservation.

## Ethics statement

The study was approved by the University of the West Indies, Cave Hill Campus/Barbados Ministry of Health, and Wellness Research Ethics Committee/Institutional Review Board (IRB No. 210322-B). The patients/participants provided their written informed consent to participate in this study.

## Author contributions

AB, KK, DC, and MM contributed to the conceptualization of the project. KK, CF, AB, MM, and RC-H contributed to the methodology and data collection. AB, KK, DC, SR, and MM wrote original draft. AR, AB, and MM analyzed the data. AB, KK, DC, NO, and MM contributed to the project administration. RK, AP, NO, and AR critically read, edited the manuscript, and provided useful suggestions on data analysis. AB, KK, and MM have full access to all the data and take responsibility for the integrity of the data. All authors have read and agreed to the published version of the manuscript.

## Conflict of interest

The authors declare that the research was conducted in the absence of any commercial or financial relationships that could be construed as a potential conflict of interest.

## Publisher's note

All claims expressed in this article are solely those of the authors and do not necessarily represent those of their affiliated organizations, or those of the publisher, the editors and the reviewers. Any product that may be evaluated in this article, or claim that may be made by its manufacturer, is not guaranteed or endorsed by the publisher.

## References

[1] Sahu AK Amrithanand VT Mathew R Aggarwal P Nayer J Bhoi S. COVID-19 in health care workers - a systematic review and meta-analysis. Am J Emerg Med. (2020) 38:1727–31. 10.1016/j.ajem.2020.05.113 32738467 PMC7837172

[2] Krishnamurthy K Sobers N Kumar A Ojeh N Scott A Cave C. COVID-19 vaccine intent among health care professionals of Queen Elizabeth Hospital, Barbados. J Multidiscip Healthc. (2021) 14:3309–19. 10.2147/JMDH.S336952 34876817 PMC8643144

[3] Alam A Azim Majumder MA Haque M Ashraf F Khondoker MU Mashreky SR. Disproportionate COVID-19 vaccine acceptance rate among healthcare professionals on the eve of nationwide vaccine distribution in Bangladesh. Expert Rev Vaccines. (2021) 20:1167–75. 10.1080/14760584.2021.1951248 34224292

[4] Ashok N Krishnamurthy K Singh K Rahman S Majumder MAA. High COVID-19 vaccine hesitancy among healthcare workers: should such a trend require closer attention by policymakers? Cureus. (2021) 13:e17990. 10.7759/cureus.17990 34667668 PMC8519358

[5] Majumder MAA Lutfor AB Rabbi AMF Alam A Rahman M Saha N. Prevalence of COVID-19 vaccine reactogenicity among Bangladeshi physicians. FASEB Bioadv. (2022) 4:379–90. 10.1096/fba.2021-00158 35601057 PMC9111157

[6] Peeri NC Shrestha N Rahman MS Zaki R Tan Z Bibi S. The SARS, MERS and novel coronavirus (COVID-19) epidemics, the newest and biggest global health threats: what lessons have we learned? Int J Epidemiol. (2020) 49:717–26. 10.1093/ije/dyaa033 32086938 PMC7197734

[7] Hsin DH Macer DR. Heroes of SARS: professional roles and ethics of health care workers. J Infect. (2004) 49:210–5. 10.1016/j.jinf.2004.06.005 15337337 PMC7132465

[8] Alhazzani W Møller MH Arabi YM Loeb M Gong MN Fan E. Surviving sepsis campaign: guidelines on the management of critically ill adults with Coronavirus Disease 2019 (COVID-19). Intens Care Med. (2020) 46:854–87. 10.1007/s00134-020-06022-5 32222812 PMC7101866

[9] Cook TM. Personal protective equipment during the coronavirus disease (COVID) 2019 pandemic - a narrative review. Anaesthesia. (2020) 75:920–7. 10.1111/anae.15071 32246849

[10] Guidance for the Selection and Use of Personal Protective Equipment (PPE) in Healthcare Settings. Department of Health and Human Services Centers for Disease Control and Prevention (2021). Available online at: https://www.cdc.gov/hai/pdfs/ppe/ppeslides6-29-04.pdf.

[11] Rational Use of Personal Protective Equipment for Coronavirus Disease (COVID-19) and Considerations During Severe Shortages. World Health Organisation (2020).

[12] Cheng VC Wong SC Chuang VW So SY Chen JH Sridhar S. The role of community-wide wearing of face mask for control of coronavirus disease 2019 (COVID-19) epidemic due to SARS-CoV-2. J Infect. (2020) 81:107–14. 10.1016/j.jinf.2020.04.024 32335167 PMC7177146

[13] Honda H Iwata K. Personal protective equipment and improving compliance among healthcare workers in high-risk settings. Curr Opin Infect Dis. (2016) 29:400–6. 10.1097/QCO.0000000000000280 27257793

[14] Hoernke K Djellouli N Andrews L Lewis-Jackson S Manby L Martin S. Frontline healthcare workers' experiences with personal protective equipment during the COVID-19 pandemic in the UK: a rapid qualitative appraisal. BMJ Open. (2021) 11:e046199. 10.1136/bmjopen-2020-046199 33472794 PMC7818840

[15] Galanis P Vraka I Fragkou D Bilali A Kaitelidou D. Impact of personal protective equipment use on health care workers' physical health during the COVID-19 pandemic: a systematic review and meta-analysis. Am J Infect Control. (2021) 49:1305–15. 10.1016/j.ajic.2021.04.084 33965463 PMC8102386

[16] Jose S Cyriac MC Dhandapani M. Health problems and skin damages caused by personal protective equipment: experience of frontline nurses caring for critical COVID-19 patients in intensive care units. Indian J Crit Care Med. (2021) 25:134–9. 10.5005/jp-journals-10071-23713 33707889 PMC7922454

[17] Abiakam N Worsley P Jayabal H Mitchell K Jones M Fletcher J. Personal protective equipment related skin reactions in healthcare professionals during COVID-19. Int Wound J. (2021) 18:312–22. 10.1111/iwj.13534 33507634 PMC8013193

[18] Park C-Y KK Roth S Beck S Kang JW Tayag MC. Global shortage of personal protective equipment amid COVID-19: supply chains, bottlenecks, and policy implications. Asian Dev Bank. (2020) 130:1–10. 10.22617/BRF200128-2

[19] Cohen J Rodgers YVM. Contributing factors to personal protective equipment shortages during the COVID-19 pandemic. Prev Med. (2020) 141:106263. 10.1016/j.ypmed.2020.106263 33017601 PMC7531934

[20] Zhou NY Yang L Dong LY Li Y An XJ Yang J. Prevention and treatment of skin damage caused by personal protective equipment: experience of the first-line clinicians treating 2019-nCoV infection. Int J Dermatol Venereol. (2020) 3:70–75. 10.1097/JD9.0000000000000085 34192087 PMC7147274

[21] Hu K Fan J Li X Gou X Li X Zhou X. The adverse skin reactions of health care workers using personal protective equipment for COVID-19. Medicine. (2020) 99:e20603. 10.1097/MD.0000000000020603 32541493 PMC7302613

[22] Yu J Goldminz A Chisolm S Jacob SE Zippin JH Wu PA. Facial personal protective equipment: materials, resterilization methods, and management of occupation-related dermatoses. Dermatitis. (2021) 32:78–85. 10.1097/DER.0000000000000699 33273243

[23] Coronavirus in Barbados. Covid Observer. (2021). Available online at: https://covid.observer/bb/ (accessed November 25, 2021).

[24] Hakim M Khattak FA Muhammad S Ismail M Ullah N Atiq Orakzai M. Access and use experience of personal protective equipment among frontline healthcare workers in Pakistan during the COVID-19 emergency: a cross-sectional study. Health Secur. (2021) 19:140–9. 10.1089/hs.2020.0142 33175583

[25] Liu Q Luo D Haase JE Guo Q Wang XQ Liu S. The experiences of health-care providers during the COVID-19 crisis in China: a qualitative study. Lancet Glob Health. (2020) 8:e790–8. 10.1016/S2214-109X(20)30204-7 32573443 PMC7190296

[26] Iwu CJ Jordan P Jaca A Iwu CD Schutte L Wiysonge CS. Cochrane corner: personal protective equipment for preventing highly infectious diseases such as COVID-19 in healthcare staff. Pan Afr Med J. (2020) 37:148. 10.11604/pamj.2020.37.148.24934 33425181 PMC7757305

[27] Metin N Turan Ç Utlu Z. Changes in dermatological complaints among healthcare professionals during the COVID-19 outbreak in Turkey. Acta Dermatovenerol Alp Pannonica Adriat. (2020) 29:115–22. 10.15570/actaapa.2020.25 32975297

[28] Çiriş Yildiz C Ulaşli Kaban H Tanriverdi F. COVID-19 pandemic and personal protective equipment: evaluation of equipment comfort and user attitude. Arch Environ Occup Health. (2020) 77:1–8. 10.1080/19338244.2020.1828247 33063614

[29] Lin P Zhu S Huang Y Li L Tao J Lei T. Adverse skin reactions among healthcare workers during the coronavirus disease 2019 outbreak: a survey in Wuhan and its surrounding regions. Br J Dermatol. (2020) 183:190–2. 10.1111/bjd.19089 32255197 PMC7262186

[30] Green S Gani A Bailey M Brown O Hing CB. Fit-testing of respiratory protective equipment in the UK during the initial response to the COVID-19 pandemic. J Hosp Infect. (2021) 113:180–6. 10.1016/j.jhin.2021.04.024 33940089 PMC8087583

[31] Lan J Song Z Miao X Li H Li Y Dong L. Skin damage among health care workers managing coronavirus disease-2019. J Am Acad Dermatol. (2020) 82:1215–6. 10.1016/j.jaad.2020.03.014 32171808 PMC7194538

[32] Daye M Cihan FG Durduran Y. Evaluation of skin problems and dermatology life quality index in health care workers who use personal protection measures during COVID-19 pandemic. Dermatol Ther. (2020) 33:e14346. 10.1111/dth.14346 32985745 PMC7536955

[33] Kiely LF Moloney E O'Sullivan G Eustace JA Gallagher J Bourke JF. Irritant contact dermatitis in healthcare workers as a result of the COVID-19 pandemic: a cross-sectional study. Clin Exp Dermatol. (2021) 46:142–4. 10.1111/ced.14397 32705718 PMC7404516

[34] Gefen A. Reswick and Rogers pressure-time curve for pressure ulcer risk. Part 1. Nurs Stand. (2009) 23:64:6, 8 passim. 10.7748/ns2009.07.23.45.64.c7115 19678520

[35] Grap MJ Munro CL Wetzel PA Schubert CM Pepperl A Burk RS. Tissue interface pressure and skin integrity in critically ill, mechanically ventilated patients. Intensive Crit Care Nurs. (2017) 38:1–9. 10.1016/j.iccn.2016.07.004 27836262 PMC5641974

[36] Jiang Q Song S Zhou J Liu Y Chen A Bai Y. The prevalence, characteristics, and prevention status of skin injury caused by personal protective equipment among medical staff in fighting COVID-19: a multicenter, cross-sectional study. Adv Wound Care. (2020) 9:357–64. 10.1089/wound.2020.1212 32320359 PMC7307701

[37] Yan Y Chen H Chen L Cheng B Diao P Dong L. Consensus of Chinese experts on protection of skin and mucous membrane barrier for health-care workers fighting against coronavirus disease (2019). Dermatol Ther. (2020) 33:e13310. 10.1111/dth.13310 32170800 PMC7228211

[38] Darlenski R Tsankov N. COVID-19 pandemic and the skin: what should dermatologists know? Clin Dermatol. (2020) 38:785–7. 10.1016/j.clindermatol.2020.03.012 33341217 PMC7102542

[39] Keng BMH Gan WH Tam YC Oh CC. Personal protective equipment-related occupational dermatoses during COVID-19 among health care workers: a worldwide systematic review. JAAD Int. (2021) 5:85–95. 10.1016/j.jdin.2021.08.004 34485949 PMC8407949

[40] Yu J Chen JK Mowad CM Reeder M Hylwa S Chisolm S. Occupational dermatitis to facial personal protective equipment in health care workers: a systematic review. J Am Acad Dermatol. (2021) 84:486–94. 10.1016/j.jaad.2020.09.074 33011325 PMC7528888

[41] Battista RA Ferraro M Piccioni LO Malzanni GE Bussi M. Personal Protective Equipment (PPE) in COVID 19 pandemic: related symptoms and adverse reactions in healthcare workers and general population. J Occup Environ Med. (2021) 63:e80–5. 10.1097/JOM.0000000000002100 33298757 PMC7864606

[42] Foo CC Goon AT Leow YH Goh CL. Adverse skin reactions to personal protective equipment against severe acute respiratory syndrome–a descriptive study in Singapore. Contact Dermatitis. (2006) 55:291–4. 10.1111/j.1600-0536.2006.00953.x 17026695 PMC7162267

[43] Lim EC Seet RC Lee KH Wilder-Smith EP Chuah BY Ong BK. Headaches and the N95 face-mask amongst healthcare providers. Acta Neurol Scand. (2006) 113:199–202. 10.1111/j.1600-0404.2005.00560.x 16441251 PMC7159726

[44] Williams WJ Cichowicz JK. Heat Stress Imposed by PPE Worn in Hot and Humid Environments. CDC (2020). Available online at: https://blogs.cdc.gov/niosh-science-blog/2020/08/06/ppe-heat-stress/ (accessed January 8, 2022).

[45] Yuan X Xi H Le Y Xu H Wang J Meng X. Online survey on healthcare skin reactions for wearing medical-grade protective equipment against COVID-19 in Hubei Province, China. PLoS ONE. (2021) 16:e0250869. 10.1371/journal.pone.0250869 33914813 PMC8084174

[46] Regli A Sommerfield A von Ungern-Sternberg BS. The role of fit testing N95/FFP2/FFP3 masks: a narrative review. Anaesthesia. (2021) 76:91–100. 10.1111/anae.15261 32932556

[47] Hu D Kong Y Li W Han Q Zhang X Zhu LX. Frontline nurses' burnout, anxiety, depression, and fear statuses and their associated factors during the COVID-19 outbreak in Wuhan, China: a large-scale cross-sectional study. EClinicalMedicine. (2020) 24:100424. 10.1016/j.eclinm.2020.100424 32766539 PMC7320259

[48] Dhandapani M Dhandapani S. Challenges posed by COVID-19 and neurosurgical nursing strategies in developing countries. Surg Neurol Int. (2020) 11:441. 10.25259/SNI_677_2020 33408926 PMC7771480

[49] Dubaniewicz MT Rottach DR Yorio PL. Quality assurance sampling plans in US stockpiles for personal protective equipment: a computer simulation to examine degradation rates. Health Secur. (2019) 17:324–33. 10.1089/hs.2019.0042 31433277 PMC6823634

[50] LeBlanc KCH Butt B Bresnai-Harris J Wiesenfeld L. Prevention and Management of Skin Damage Related to Personal Protective Equipment (PPE): Hospital News, Canada's Health Care News and Best Practices, Vertical Media. Available online at: https://hospitalnews.com/prevention-and-management-of-skin-damage-related-to-personal-protective-equipment-ppe/ (accessed January 8, 2022).

[51] Gefen A Ousey K. Update to device-related pressure ulcers: SECURE prevention. COVID-19, face masks and skin damage. J Wound Care. (2020) 29:245–59. 10.12968/jowc.2020.29.5.245 32421479

