# Supplementary material for: Personal protective equipment (PPE) related adverse skin reactions among healthcare workers at the main COVID-19 isolation center in Barbados
Source: Front Public Health. 2022 Oct 11;10:978590. doi: 10.3389/fpubh.2022.978590 (PMC9592812; doi:10.3389/fpubh.2022.978590)
Supplement: Supplementary file 1 [file Data_Sheet_1.PDF]

Personal protective equipment related adverse skin reactions among healthcare professionals at COVID-19 Isolation centres

Thank you very much for your interest in this survey. This study aimed to assess the personal protective equipment-related adverse skin reactions among healthcare professionals at COVID-19 Isolation centres. A healthcare professional is one who delivers care and services to the sick and ailing. Healthcare workers will participate in the survey. The questionnaire will take approximately 15- 20 minutes.

Please read the consent form, and if you agree to participate, click YES in the box at the end of the form.

*Required

1. I confirm that I am a healthcare professional at COVID-19 Isolation centre: *

   Mark only one oval.

   - Yes
   - No

Personal protective equipment related adverse skin reactions among healthcare professional

2. I HAVE READ THIS DESCRIPTION AND CONSENT TO PARTICIPATE: *

   Mark only one oval.

   - Yes
   - No

Personal protective equipment related adverse skin reactions among healthcare professional

3. Age *

   Mark only one oval.

   - 18 - 24
   - 25 - 34
   - 35 - 44
   - 45 - 54
   - 55+

4. Gender *

   Mark only one oval.

   - Male
   - Female
   - Transgender
   - Non-binary
   - Prefer not to say
   - Other: ____________

5. Nationality *

Mark only one oval.

- ◯ Barbados
- ◯ Jamaica
- ◯ Trinidad and Tobago
- ◯ Cuba
- ◯ India
- ◯ USA
- ◯ Other: ____________________

6. What is your current role during COVID-19? *

Mark only one oval.

- ◯ Nurse
- ◯ Doctor
- ◯ Orderly
- ◯ Housekeeping
- ◯ Other: ____________________

7. On average, how many days do you work in a week? *

____

PPE FOR EYES AND FACE PROTECTION

8. What type of eye protection are you using when attending your duties? *

Mark only one oval.

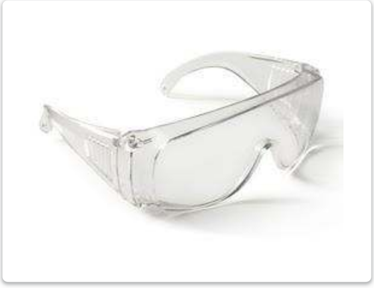

◯ General safety glasses

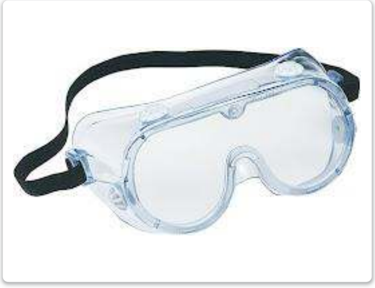

◯ Chemical splashing goggles

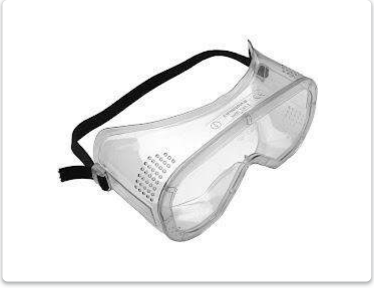

◯ Impact goggles

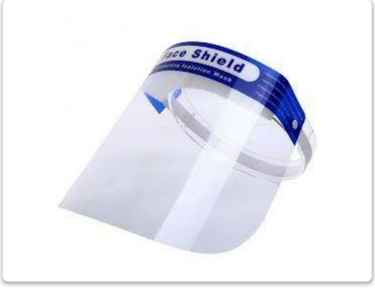

◯ Face shield

◯ Other: __________

9. What type of face protection are you using when attending your duties? *

Mark only one oval.

- Surgical Mask
- N95
- K95
- Both
- Other: ____________________

10. Did you attend any fit testing appointment before adopting eye and face protection equipment? *

Mark only one oval.

- Yes
- No

11. How long you are using PPE? *

Mark only one oval.

- 0 - less than 3 months
- 3 - less than 6 months
- 6 - less than 9 months
- 9 - less than 12 months
- >1 year

12. On average, how many hours do you wear this protective equipment in a day? *

Mark only one oval.

- less than 6
- 6-8
- 8-10
- 10-12
- 12-13
- Other: ____________________

13. Do you wear this protective equipment for consecutive days? *

Mark only one oval.

- Yes
- No

14. If yes, what is the maximum number of consecutive days? *

Mark only one oval.

- Every 2 days
- Every 3 days
- Every 4 days
- Every 5 days
- >5 days

HOW COMFORTABLE IS THE PPE

15. Please select your answer *

Mark only one oval per row.

	Yes	No
Do you feel comfortable wearing this protective equipment?		
Are you able to breathe easily when wearing this protective equipment?		
Do you feel safe and in control when wearing this protective equipment?		

16. If 0 is no pain and 10 is the worse pain imaginable, can you rate your pain, when wearing PPE, on the scale below:

Mark only one oval.

	0	1	2	3	4	5	6	7	8	9	10	
No Pain												The worse pain imaginable

FACE SKIN HEALTH

17. Please select your answer

Mark only one oval per row.

	Yes	No
Do you have any red marks where the protective equipment is placed?		
Do you have any indentation marks where the protective equipment is placed?		
Is your skin broken on the area where the protective equipment is placed?		

18. Using the face diagram, please annotate the sites of your skin presenting with redness, itchiness, rash or pressure damage due to the use of PPE:

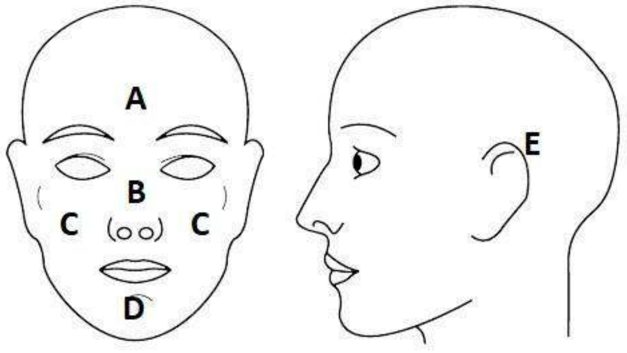

Tick all that apply.

	Redness blanching	Itchiness	Rash	Pressure damage	Spots	Dry Skin	No Reaction
Site A	☐	☐	☐	☐	☐	☐	☐
Site B	☐	☐	☐	☐	☐	☐	☐
Site C	☐	☐	☐	☐	☐	☐	☐
Site D	☐	☐	☐	☐	☐	☐	☐
Site E	☐	☐	☐	☐	☐	☐	☐

19. How good or bad did you perceive the health of your face skin BEFORE STARTINGUSING PROTECTIVE EQUIPMENT

Mark only one oval.

	1	2	3	4	5	6	7	8	9	10	
The worst skin health you can imagine	◯	◯	◯	◯	◯	◯	◯	◯	◯	◯	The best skin health you can imagine

20. We would like to know how good or bad you perceive skin health TODAY

Mark only one oval.

	1	2	3	4	5	6	7	8	9	10	
The worst skin health you can imagine	◯	◯	◯	◯	◯	◯	◯	◯	◯	◯	The best skin health you can imagine

21. Would you be happy for us to monitor the health of your skin on a weekly basis using a very brief follow-up questionnaires?

Mark only one oval.

◯ Yes, please

◯ No, please

This content is neither created nor endorsed by Google.

Google Forms
